# Supplementary material for: Complexome profiling of the Chlamydomonas psb28 mutant reveals TEF5 as an early PSII assembly factor
Source: Plant Cell. 2025 Mar 17;37(6):koaf055. doi: 10.1093/plcell/koaf055 (PMC12164590; doi:10.1093/plcell/koaf055)
Supplement: koaf055_Supplementary_Data [file koaf055_supplementary_data.zip › psb28_interactive.zip!/plots/intensity/qualityPlots/peptides/_Cre01.g014000.t1.2.html]

### Cre01.g014000.t1.2

Trivial name:   
SeqCoverage [median]: 59.70  
Mass: 20.95 kDa  
  
 

| Sequence | MissedCleavage | Mass [Da] | wtIntensity | muIntensity | Score |
| --- | --- | --- | --- | --- | --- |
| APAAASPFGAAPAASPFGSSTGGAVGGGAGASPFK | 0 | 2945.43 | 895.20 | 3087.55 | 91.75 |
| APAAASPFGAAPAASPFGSSTGGAVGGGAGASPFKR | 1 | 3101.53 | 4564.50 | 5242.77 | 133.94 |
| DGPTISANTPNPFGK | 0 | 1514.74 | 20779.43 | 33797.23 | 237.67 |
| DIEPAGLRPDMSPDPFVK | 1 | 1982.98 | 16296.05 | 36100.54 | 79.55 |
| DIEPAGLRPDMSPDPFVKETPVSMLK | 2 | 2868.44 | 3093.65 | 2379.25 | 40.94 |
| DRKEFLGNAQNDILEK | 2 | 1888.96 | 283.42 | 719.07 | 56.41 |
| EFLGNAQNDILEK | 0 | 1489.74 | 7397.82 | 15223.75 | 186.02 |
| ETPVSMLK | 0 | 903.47 | 0.00 | 409.01 | 79.47 |
| IWGAEEENAAQVR | 0 | 1471.71 | 566.40 | 0.00 | 71.35 |
| KEFLGNAQNDILEK | 1 | 1617.84 | 0.00 | 459.54 | 74.30 |
| YKVELGDAVEVAPK | 1 | 1516.81 | 10341.75 | 24507.35 | 122.78 |
|  |  |  | 64218.2 | 121926.1 |  |
